# Supplementary material for: Local mapping of the nanoscale viscoelastic properties of fluid membranes by AFM nanorheology
Source: Nat Commun. 2025 Apr 24;16:3842. doi: 10.1038/s41467-025-59260-w (PMC12019565; doi:10.1038/s41467-025-59260-w)
Supplement: Supplementary file 1 — Supplementary Information [file 41467_2025_59260_MOESM1_ESM.pdf]

Supplementary information

Local mapping of the nanoscale viscoelastic properties of fluid membranes by AFM nanorheology

William J. Trewby[1,†,*], Mahdi Tavakol[1,‡], Kislon Voïtchovsky[1,*]

[1] Physics Department, Durham University, South Road, Durham, DH1 3LE, UK
[†] present address: London Centre for Nanotechnology, University College London, London, WC1H 0AH, UK
[‡] present address: Department of Engineering Science, University of Oxford, Parks Road, Oxford OX1 3PJ, UK

email: william.trewby.10@ucl.ac.uk, kislon.voitchovsky@durham.ac.uk

Contents of the supplementary information

Supplementary Note 1. Diffusion coefficients of supported lipid membranes ... 2

Supplementary Note 1.1. Examples of literature values of *D* for supported lipid membranes in solution ... 2

Supplementary Note 1.2. Physical model for calculating *D* from shear the force spectroscopy measurements ... 3

Supplementary Note 1.3. Demonstrating local correlations in diffusion coefficients ... 4

Supplementary Note 2. Assembly and calibration of the nano-shearing device ... 6

Supplementary Note 2.1. Calibration ... 6

Supplementary Note 2.2. Optimising for high-frequency operation ... 7

Supplementary Note 3. Extraction of local storage and loss moduli for the lipids ... 11

Supplementary Note 4. Impact of tip-lipid contact area ... 13

Supplementary Note 5. Local membrane perturbation ... 15

Supplementary Methods' Figures and Table ... 16

Supplementary References ... 19

Supplementary Note 1. Diffusion coefficients of supported lipid membranes

Supplementary Note 1.1. Examples of literature values of *D* for supported lipid membranes in solution

Supplementary Table 1: Examples of diffusion coefficients of supported synthetic lipid membranes and natural biological membranes, obtained in different environments and with a range of technique. It is a non-exhaustive list and aimed as overview of the typical diffusion coefficient values reported for common lipids with different techniques, substrates and imaging solutions.

Lipid species	D ($\mu m^2\ s^{-1}$)	Solution	Substrate	Temp (°C)	Technique	Fluorophore	Source
DOPC	3.743	Water	SiO_2	25.4	FRAP	NBD-PE, NBD-DPPE	Tamm and McConnell[1]
DMPC	2.497	Water	SiO_2	24.6	FRAP	NBD-PE, NBD-DPPE	Tamm and McConnell[1]
DOPC	3.1 ± 0.3	Glucose[a]	Mica	15.9	Z-scan FCS	BODIPY-DHPE	Przybylo *et al.*[2]
DOPC	2.7 ± 0.3	Buffer[b]	Mica	15.9	Z-scan FCS	BODIPY-DHPE	Przybylo *et al.*[2]
DOPC	4.2 ± 0.4	Buffer[b]	Mica	23 (RT)	Z-scan FCS	Rhodamine Red-X DHPE	Benda *et al.*[3]
DOPC	4.0 ± 0.5	Buffer[b]	Borosilicate glass	23 (RT)	Z-scan FCS	Rhodamine Red-X DHPE	Benda *et al.*[3]
DLPC	8.0	Water	Mica	RT	FCS	NBD-PC	Ratto and Longo[4]
DLPC	2.5	Buffer[c]	Quartz	23.0	FCS	DMPE-Rhodamine B	Zhang and Granick[5]
DOPC	1.30 ± 0.15	Water	Glass[d]	RT	FRAP	Texas Red (TR)-DHPE	Zhang *et al.*[6]
Cell plasma membrane (COS-7)	2.0-2.5	"Regular cell media"	Cell	Not given	SM*d*M[e]	BDP-TMR-alkyne	Yan *et al.*[7]
Cell plasma membrane (hippocampal neuron)	≈0.5-2.0	Neurobasal medium[f]	Cell	37	STORM	DiI	Shim *et al.*[8]
DiPhyPC	≈1.2-3.0	200 mM sucrose	Glass	15-30	FCS, SPT	Texas Red (TR)-DHPE	Woodward and Kelly[9]
DOPC	≈8.2	Water[g]	Ordered multilayer lipid stack	25	PFG-NMR	N/A	Filippov *et al.*[10]

[a] 150 mOsm
[b] 10 mM HEPES; 150 mM NaCl; 2 mM $CaCl_2$
[c] 10 mM PBS
[d] Micropatterned polyelectrolyte layer also present for electrical measurements
[e] Single-molecule displacement/diffusivity mapping
[f] Supplemented with 4% of 1 M HEPES (pH 7.4) and the oxygen scavenging system
[g] Controlled hydration: 35 wt.%

Supplementary Note 1.2. Physical model for calculating *D* from shear the force spectroscopy measurements

Once the torsional motion of the cantilever has been converted into a force (see Methods), force-distance spectroscopy measurements return an absolute measure of the lateral drag force, F_S, experienced by the tip at each tip-membrane distance probed for the imposed shear frequency f_S and amplitude A_S. The use of small cantilevers and small fluid volumes ensures that F_S is finite only when the AFM tip is in contact with the lipid bilayer or the mica substrate underneath, showing that the force measured is solely due to the lipids' response to the shear. Assuming that the Einstein relation[11] is valid in this two-dimensional lipid system, the lipids' mobility, μ, is related to their diffusion coefficient D by $D = \mathrm{k_B}T\,\mu$. Without loss of generality, the mobility can be written as the ratio between the drift velocity of a particle in the fluid and the drag force experienced as it moves. If we assume the tip to be coupled to the lipids directly touching it, the drift velocity of these coupled lipids is given by the RMS velocity of the shear piezo, $v_{\mathrm{RMS}} = f_S A_S/\sqrt{2}$, and the drag force is F_S, as calculated from the torsional lever motion. This allows us to re-write the Einstein relation for the group of lipids being dragged by the tip:

$$D_{\mathrm{eff}} = \mathrm{k_B}T \cdot \mu = \mathrm{k_B}T\frac{v_{\mathrm{RMS}}}{F_S} = \mathrm{k_B}T\frac{f_S A_S}{\sqrt{2}F_S}. \qquad (1)$$

Here, the effective diffusion coefficient, D_{eff}, indicates that we are probing the dynamics of a group of lipid molecules dragged by the tip. This can be related to the diffusion coefficient of single lipid molecules using the Evans-Sackmann approximation[12,13] for the diffusion of a disc-like inclusions of radius r_{disc} within a supported lipid bilayer. The Evans-Sackmann approximation takes the Saffman-Delbrück model for diffusion within a free-standing bilayer[14] and applies it to the case where there is a variable coupling between the lipids and a fixed support. If there is strong coupling, as to be expected between the hydrophilic lipid headgroups and mica crystal, the effective diffusion coefficient, D_{eff}, of the inclusion scales with the inverse square of the disc's radius, r_{disc}:[13]

$$D_{\mathrm{eff}} \propto r_{\mathrm{disc}}^{-2}. \qquad (2)$$

If we assume the group of lipids being dragged by the tip to behave as a disk-shaped inclusion of area A_{tip}, we can then use supplementary Eq. (2) to relate the diffusion coefficient of the inclusion to that of a single lipid molecule D_{lipid} through $D_{\mathrm{lipid}} = D_{\mathrm{eff}}\,A_{\mathrm{tip}}/A_{\mathrm{lipid}}$, with A_{lipid} the area of a single lipid molecule. Typically, $A_{\mathrm{lipid}} \sim 0.8\ \mathrm{nm}^2$.[15] We take the area of the disc-like inclusion to be the tip-lipid contact area, a spherical cap depending on the tip indentation Δh in the membrane and the tip radius, R_{tip} so that $A_{\mathrm{tip}} = 2\pi R_{\mathrm{tip}}\Delta h$. Combining these results, we finally get Eq. (1) as presented in the manuscript:

$$D_{\mathrm{lipid}} = \mathrm{k_B}T \frac{2\pi R_{\mathrm{tip}}\Delta h}{A_{\mathrm{lipid}}} \frac{f_{\mathrm{S}} A_{\mathrm{S}}}{\sqrt{2}F_{\mathrm{S}}}. \quad (3)$$

Supplementary Note 1.3. Demonstrating local correlations in diffusion coefficients

The distinction between the diffusive properties and force curves of the L_O and L_D phases of the ternary membrane are evident from Fig. 3c-d, but the histogram of D values (d) clearly shows a broad spread in the data. Yet, the spread of values in any given single measurement is relatively small (Fig. 1c-e, Fig 2c). This suggest that the above-mentioned data spread is not due to experimental uncertainties but rather reflects local variations in D within each phase. To address this question statistically, we make use of a quantity known as the spatial lag, Y_{SL}, which is a measure of similarity between a data point and those which are nominally connected to it[16]. In our case, we are interested in whether the diffusion coefficient, D, at a given pixel location is correlated with those pixels around it. For random instrumental noise, we would expect there to be no relationship, whereas dynamic lipid motion is more likely to be spatially correlated. Formally, the spatial lag for a quantity Y is defined as[17]:

$$Y_{\mathrm{SL}} = WY, \quad (4)$$

where W is a matrix of spatial weights – that is, the level of connectivity between each data point. In the case of Fig. 3a-b, we constructed W so that it captured nearest neighbour behaviour within the same lipid phase (as deduced from the topography). This effectively means that each element of Y_{SL} represents a weighted average of the diffusion coefficients directly connected to the corresponding element of Y, y_{ij}, (as long as they are in the same lipid phase, Fig. 3e). We then normalise Y_{SL} by the mean diffusion coefficient measured in each phase and plot this for every pixel versus the normalised diffusion coefficient, Y (Fig. 3f). If there is spatial correlation, we expect pixels with higher diffusivities to be also connected to pixels which also have relatively high values of D, which would result in a positive correlation between the (normalised) D and corresponding Y_{SL} values. Statistically this can be captured by the quantity known as Moran's I value[16,17]:

$$I = \frac{n}{\sum_i \sum_j w_{ij}} \frac{\sum_i \sum_j w_{ij} y_i y_j}{\sum_i y_i^2}. \quad (5)$$

Here, n is the total number of data points (pixels), and the other elements are as before. Describing as it does the correlation between D and Y_{SL}, Moran's I is functionally equivalent to the calculated gradient of Fig. 3f (straight line), which we find to be 0.158 ± 0.042. This indicates that there are indeed local correlations in the nanoscale diffusivity. To quantify the statistical significance of this result and ensure

that it is not down to random chance, we perform the same calculation on a modified data set, where the normalised values of D have been randomly permuted. Repeating this 5×10^4 times and recording each value of I, we build up a probability density function, plotted in Suppl. Fig. 1 below, and fitted with a Gaussian function (red). With this we calculate the p-value of our measured correlation to be $p(I \geq 0.158) = 2.25 \times 10^{-3}$, and conclude that the measured correlation is genuinely intrinsic to the lipids and not the result of instrumental noise.

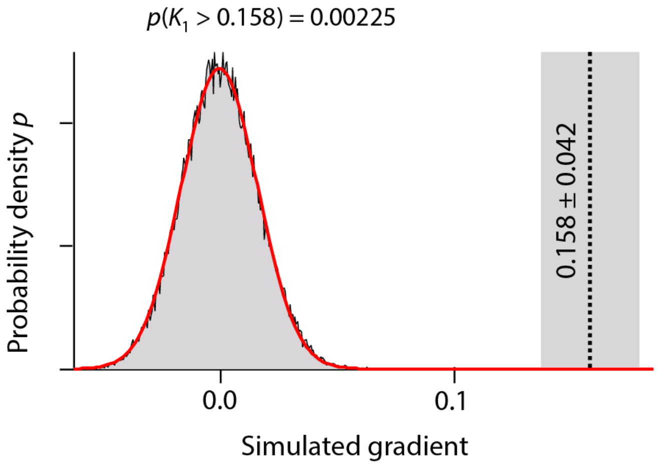

Supplementary Fig. 1 | Graphical representation of the statistical testing of the spatial correlation hypothesis. The correlated dataset (as presented in Fig. 3f) results in a Moran value of $I = 0.158 \pm 0.042$. The same dataset randomised leads to a Gaussian distribution of I values centred around zero. A P-test conducted on the random dataset confirms that our correlation is statistically highly significant given the resulting p value of 0.0025 (< 0.01, usual criterium for high significance). Source data are provided as a Source Data file.

Supplementary Note 2. Assembly and calibration of the nano-shearing device

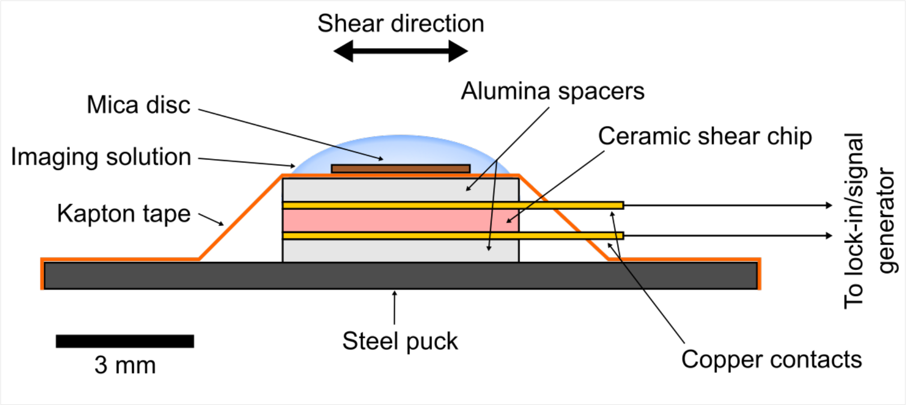

Supplementary Fig. 2 | Schematic of the shear piezo construction and components. Horizontal axis is approximately to scale. The ceramic shear chip, copper contacts and alumina spacers are purchased pre-assembled by the manufacturer.

Supplementary Note 2.1. Calibration

The shear piezo operated in an open-loop mode, and so required calibrating to know precisely the conversion between the potential applied across it and its displacement in metres. To achieve this, we mounted the device on the AFM, oriented with its direction of motion perpendicular to the long axis of the cantilever. A probe (HQ:NSC36 Cr/Au BS, MikroMasch) was then brought into contact with the freshly-cleaved mica surface in 150 mM KCl solution, with the applied load held constant at $F_{\mathrm{N}} = 5$ nN without any motion of the AFM's *x-y* scanner. Applying a low-frequency sinusoidal potential to the actuator induces a lateral motion of the sample that can be measured through the torsional motion of the cantilever. This effectively emulates lateral contact mode scanning with the shear actuator.

The slip-stick motion of the tip as it traverses the corrugated atomic lattice of the mica crystal can be clearly detected (Suppl. Fig. 3a-b) with the usual periodicity of the lattice. The experiment can be repeated as a function of the applied voltage (piezo signal amplitude). To better characterise this effect, 256 line-scans were acquired for several values of piezo amplitude. In each case, the resulting stick-slip vs voltage plot (as shown in Suppl. Fig. 3b) were analysed by fast Fourier transform to objectively identify the oscillation periodicity (Suppl. Fig. 3c). This confirmed that periodicity is due to the mica lattice parameter and not a scanning artefact. A Lorentz function was used to fit the peak location, Suppl. Fig. 3d, resulting in a lattice-voltage constant of $K_{\mathrm{lattice}} = 1.48 \pm 0.03\ \mathrm{V}^{-1}$. We then make use of the unit cell lattice parameter of the (001) plane of muscovite mica (0.519 nm) which gives us a reference length for the spacing between peaks. This leads to a final conversion factor, K_{piezo}, between applied piezo voltage and displacement of:

$$K_{\mathrm{piezo}} = 0.77 \pm 0.02\ \ \mathrm{nm} \cdot \mathrm{V}^{-1} \tag{6}$$

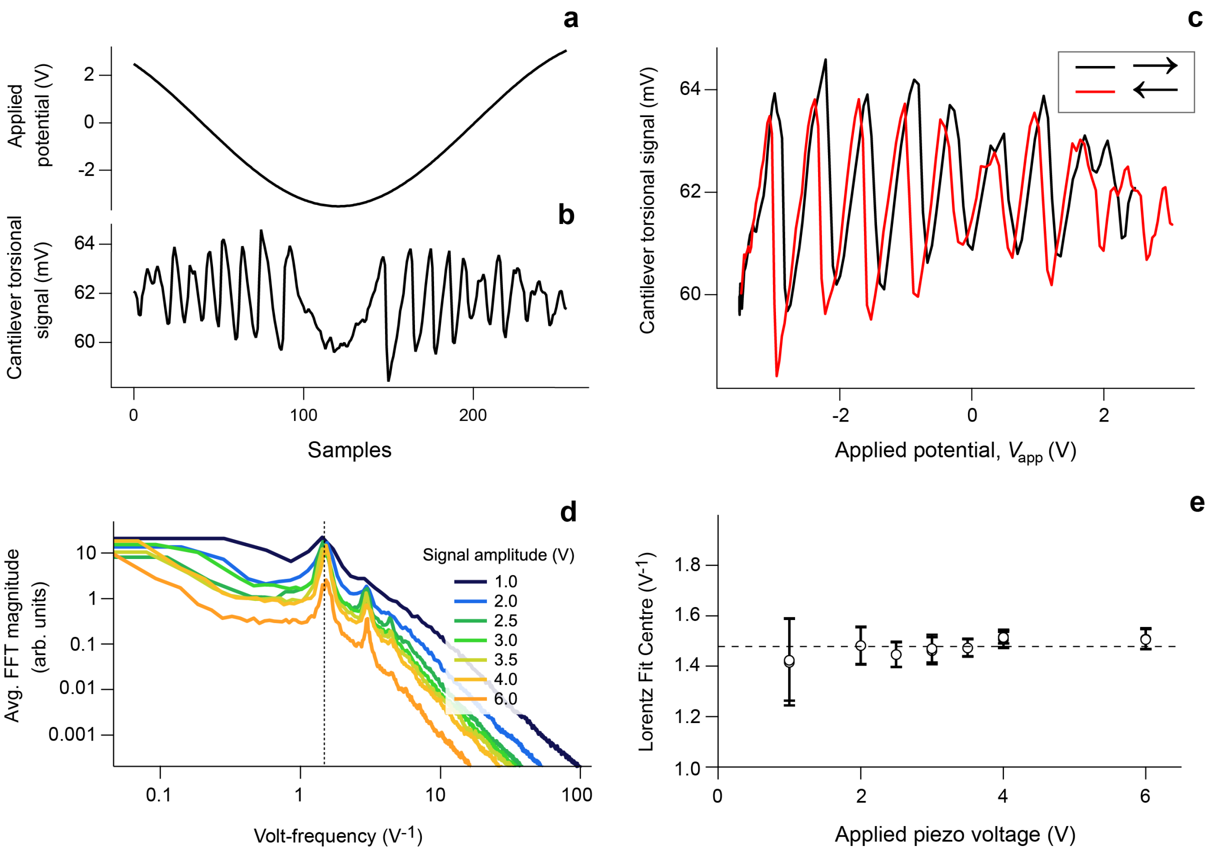

Supplementary Fig. 3 | Calibrating the shear piezo using the muscovite mica lattice constant. The shear actuator is driven by a slow sinusoidal voltage with an amplitude $|V_{app}| = 3.5$ V, (a) and made to oscillate laterally, at 90° with respect to the axis of the AFM cantilever. When the tip is in contact with the mica surface, the shear actuator operates as a lateral force microscopy scanner, revealing a stick-slip motion over the atomic corrugations of the exposed (001) mica plane (b. By plotting the measured slip-stick torsional motion of the cantilever against the instantaneous applied potential to the shear actuator (c), the periodicity of the oscillations becomes clear and is known to match the lattice parameter of mica (0.52 nm). This can be repeated for various applied potentials (i.e. piezo displacements) and systematically analysed using fast-Fourier transforms (FFT) to identify periodicity in the slip-stick motion (d). A Lorentzian fitting function is used to find the centre of each of the peaks, leading to a consistent overall average of $1.48 \pm 0.03\ \mathrm{V}^{-1}$ (e, dashed line). This translates into a calibration factor of $0.52\ \mathrm{nm} \times 1.48 \pm 0.03\ \mathrm{V}^{-1} = 0.77 \pm 0.02$ nm V^{-1}. The error bars in (e) represent the width of the Lorentzian for each fit, with $n = 2$ measurements for each voltage except 4 V where $n = 4$. Source data are provided as a Source Data file.

Supplementary Note 2.2. Optimising for high-frequency operation

In manner analogous to operating conventional AFM imaging at high-speeds[18–20], a number of mechanical challenges had to be addressed before we could perform shear force spectroscopy at sufficiently high frequencies to uncover lipid dynamics. The first is the linearity of the actuator: as the applied signal's frequency is increased and the piezoelectric crystal's resonance frequency is approached, the phase angle deviates from its ideal value of −90° and there is no longer a linear relationship between the applied potential and the displacement. We characterised our actuator using

the impedance analyser option of our lock-in amplifier (MFIA) and present the results in Suppl. Fig. 4 for both a free (red) and mounted (blue) piezo. The deviation from ideal capacitive behaviour ($\theta = -90°$; $|Z| \propto -\nu$) is evident above ≈10^5 Hz, implying we can safely operate with signals in the tens of kHz (albeit well below the nominal resonance of 1.9 MHz).

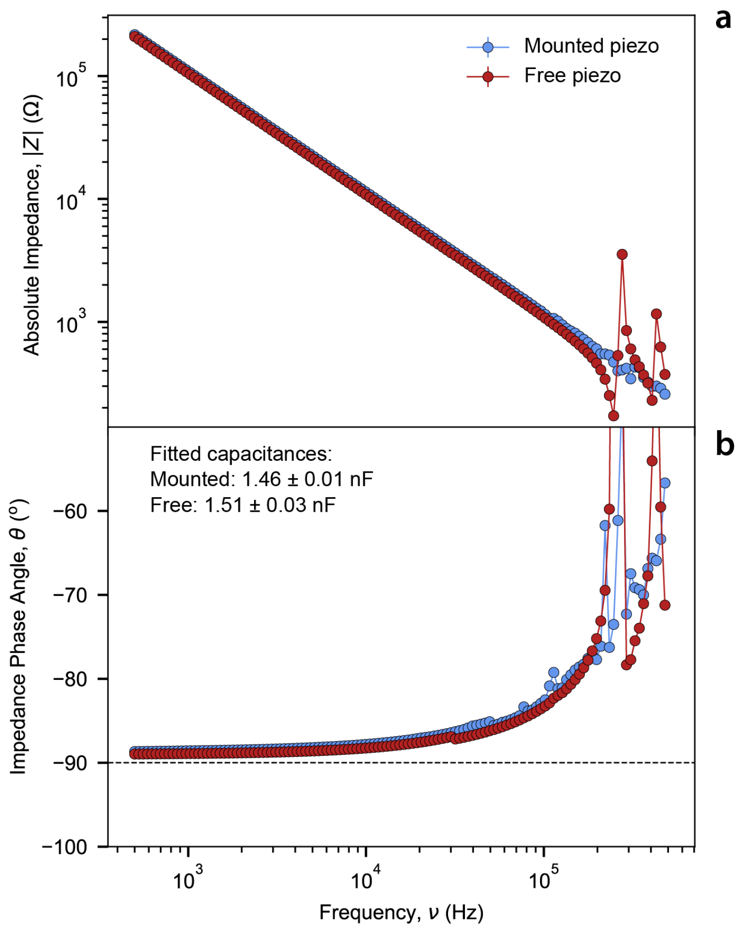

Supplementary Fig. 4 | Impedance characterisation of the shear piezo | Bode plot of the absolute impedance (a) and phase lag (b) of the shear piezo oscillating freely (red) and mounted on a steel puck (blue). The device displays capacitive behaviour up to ≈10^5 Hz, as expected. Fits to the absolute impedance demonstrate good agreement with the nominal capacitance values from the manufacturer (1.6 nF ± 15%), and mounting the piezo results in no large differences in the lower frequency (<10^5 Hz) range. Source data are provided as a Source Data file.

A second consideration that limits the dynamic capabilities of our device is the inertia of the system: as the total coupled mass (cantilever, liquid and sample) being driven increases, the resonance frequency is reduced[21] and the resultant motion becomes ill-defined and noisy, in a similar manner to the well-known 'forest-of-peaks' when driving cantilevers acoustically through an external piezo[22]. This is exemplified in Suppl. Fig. 5, in which we operate the system with a larger mica disc (12 mm diameter; grade V-1 SPI Supplies, PA, USA), a cantilever of conventional dimensions (110 × 32.5 μm^2; MikroMasch HQ:NSC36/Cr-Au BS; Apex Probes Ltd., Bracknell, UK) and a fluid droplet of containing ≈10 times the volume of liquid used for the experiments (≈50 μL).

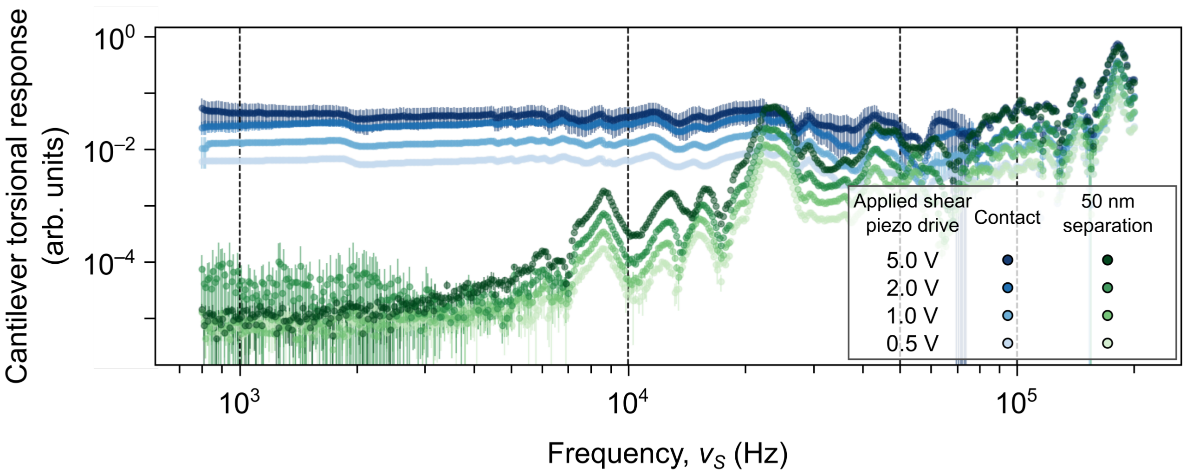

Supplementary Fig. 5 | Operating the shear device with conventional cantilevers and large fluid masses results in strong tip-sample coupling across the fluid. The shear amplitude of the cantilever, (HQ:NSC36/Au BS) is recorded by the lock-in amplifier as the shear frequency is swept from 0.8-200 kHz while the tip is either held in contact (blue shades) or at a constant separation of 50 nm (green shades) from a mica surface in ultrapure water. In contact with the mica, the interaction is relatively flat with frequency and increases with the magnitude of the shear signal from 0.5 V (light blue) to 5.0 V (navy). At a tip-mica separation of 50 nm, there is no signal at low frequencies, as expected. However, above ≈8 kHz, a clear signal can be observed for all signal strengths that eventually (at ≈20 kHz) is of the same magnitude as the signal in contact. This reflects the strong acoustic coupling of the system through the fluid when using conventional cantilevers and large droplets, even for small (nm) shear piezo oscillations. Each data point and its error is directly provided by the lock in amplifier (MFLI, Zurich Instruments) and represents a time average over each frequency (bandwidth frequency-dependent). Source data are provided as a Source Data file.

The system is solely composed of ultrapure water and mica; there are no lipids present. Frequency sweeps (or chirps) as measured by the torsional response of the cantilever, (proportional to F_L) were acquired for different applied piezo voltages, with the tip either in contact with the mica or hovering 50 nm above the surface. In contact (blue shades), the response is essentially constant with the apparition of some resonant behaviour for the higher frequencies. There is a positive correlation of the torsional response with the imposed drive on the actuator, but the absence of frequency dependence indicates that it is likely related to the absolute distance the tip is sliding relative to the mica lattice and not a velocity-dependence as for the measurements on lipids. Ideally, when the tip is separated from the substrate (green shades), the response should only display measurement noise, as indeed the case for frequencies ≲8kHz. However, there is a frequency dependence, with the torsional motion reaching values comparable to that obtained in contact measurements above ≈20 kHz. This tip-sample coupling over nanometres of fluid is analogous to amplitude-modulation AFM operated acoustically, where mechanical resonances are inevitable artefacts. This is arguably the largest limitation of the setup to lower frequencies if unaddressed.

We circumvent these mechanical resonances by reducing the total oscillating mass as much as possible.

This includes reducing the mica disc size to 3 mm and consequently the liquid mass to around 5 µL, also making use of ultra-short cantilevers (see Methods). Chirp results obtained with this updated configuration are shown in Suppl. Fig. 6. Broadly similar features as in Suppl. Fig. 5 can be observed when the tip is in contact with the mica substrate, but the measured signal with a separation of 50 nm is now close to the noise floor for all frequencies. This confirms the device's ability to perform quantitative nano-rheology at high frequencies, enabling the direct measurement of the supported lipids' diffusion coefficient, as presented in the main text.

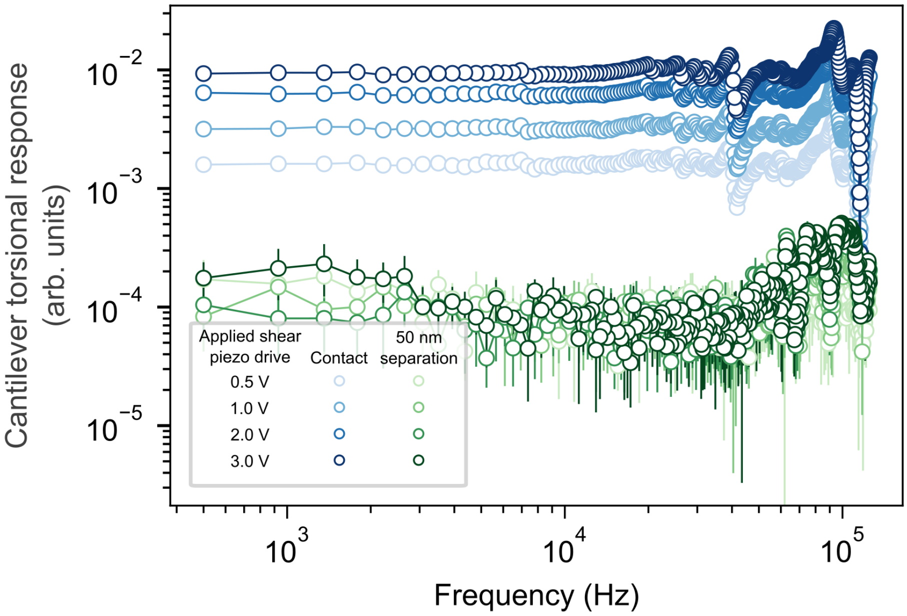

Supplementary Fig. 6 | The use of ultra-small cantilevers and low-volume fluid droplets effectively removes tip-sample coupling | Equivalent plot to Supplementary Fig. , but using ultra small cantilevers (USC-F1.5-k0.6) of nominal planar dimensions 7×3 µm and imaging solutions of just ≈5 µL. As above, there is a relatively flat torsional response when the cantilever is in contact with the mica (blue shades) and the frequency is swept (with a similar dependence on the shear signal's magnitude). However, the reduced mass and minimal planar area of the lever mean that, when the tip is separated from the substrate by 50 nm (green shades, the signal is essentially in the noise level for the entire frequency range observed here. Each data point and its error is directly provided by the lock in amplifier (MFLI, Zurich Instruments) and represents a time average over each frequency (bandwidth frequency-dependent). Source data are provided as a Source Data file.

Supplementary Note 3. Extraction of local storage and loss moduli for the lipids

From the shear force and phase, it is possible to readily calculate the storage (elastic) and loss (viscous or dissipative) moduli values for every indentation point in any given spectroscopy curve[23–25], in a same manner as for the diffusion coefficient. This is illustrated here (Suppl. Fig. 7) for the different test bilayers studied in Fig. 1.

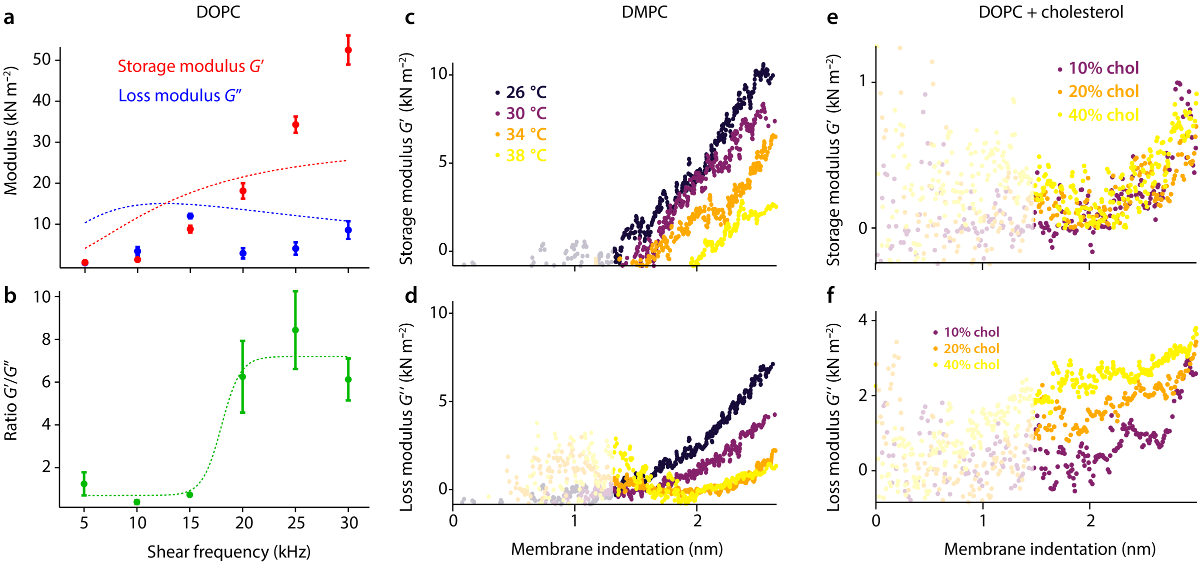

Supplementary Fig. 7: Locally measured storage and loss moduli for supported lipid bilayers | The storage (red) and loss (blue) moduli extracted over a DOPC bilayer (a) are directly obtained from the phase and amplitude of shearing[23–25]. The experimental data points have been obtained by averaging over the first 2 nm of indentation for n = 15 curves (5 kHz), n = 17 (10 kHz), n = 15 (15 kHz), n = 16 (20 kHz), n = 19 (25 kHz), n = 15 (30 kHz). In each case the error bars representing the standard deviation of the average on both the indentation and the curves. The Fitting the experimental data with a Maxwell model (dashed line) yields poor results. However, the ratio of the storage/loss moduli (b) reveals a clear and model-agnostic transition between 15 kHz and 20 kHz. The transition can be fitted with a sigmoid (dashed line) yielding a relaxation timescale of $\tau = 55 \pm 5$ µs for the area of lipids probed. The error bars are simply derived from the calculation over (a). Over DMPC, the storage (c) and loss (d) moduli are shown as a function of indentation for different temperatures. Both moduli decrease with increasing temperature for a given indentation, reflecting a fluidification of the membrane. For DOPC with increasing concentrations of cholesterol (e-f), the storage modulus (e) is too noisy to identify any trend, likely due to the storage modulus being dominated by interfacial water. The associated loss modulus (f) exhibits the expected trend with the less fluid membrane (higher cholesterol content) exhibiting the highest value due to increased viscosity. Source data are provided as a Source Data file.

The moduli capture the dynamics of both the lipids and the interfacial water, resulting in a non-negligible elastic response expected to increase with frequency. This is verified for pure DOPC bilayer

(Suppl. Fig. 7a), with the storage modulus dominating beyond ≈15 kHz due to the interfacial water. In principle, a characteristic relaxation time τ of the system can be extracted from the moduli, coinciding with the crossover from loss-dominated (lower frequencies) to storage-dominated (higher frequencies). Assuming a simple Maxwell model for the membrane, τ can be directly obtained from the ratio of the moduli[23,25]. Here, fitting the experimental results with a Maxwell model (dashed lines in Suppl. Fig. 7a) yields poor results, suggesting a more complex behaviour at play. However, the ratio of storage/loss exhibits a clear transition between 15 kHz and 20 kHz (sigmoid fit in Suppl. Fig. 7b) revealing a model-agnostic relaxation value of $\tau = 55 \pm 5$ μs for the area of lipids and interfacial water probed. The error bars represent two standard deviations over the indenting region. The storage and loss moduli can also be calculated as a function of the tip indentation into the membrane, illustrated here for DMPC at different temperatures (Suppl. Fig. 7c-d) and for DOPC membranes containing different concentrations of cholesterol. In DMPC, both the storage (Suppl. Fig. 7c) and loss (Suppl. Fig. 7d) moduli tend to decrease as the temperature increases and the membrane becomes more fluid. For the DOPC+cholesterol mixtures, the large experimental noise precludes identifying any trend for the storage modulus within error (Suppl. Fig. 7e), likely due to interfacial water dominating the measurement. However, the expected trend is well visible for the loss modulus which better reflects the viscosity of the membrane (Suppl. Fig. 7f).

Supplementary Note 4. Impact of tip-lipid contact area

Our model for the lipids' diffusion coefficient implies a straightforward dependence on the effective tip radius, R_{tip}. However, the apparently linear relationship of Suppl. Eq. (3) belies the fact that both F_L and Δh are also, for a given applied load, functions of R_{tip}. We use two approaches to assess this rather subtle dependency: a direct evaluation *via* scanning electron microscopy (SEM) of the tips used over multiple experiments (with a fresh tip used each time) to determine the impact of the experiments on the tip radius; and measurements of the normal and lateral force before and after a deliberate tip blunting procedure. An example is shown in Suppl. Fig. 8 below.

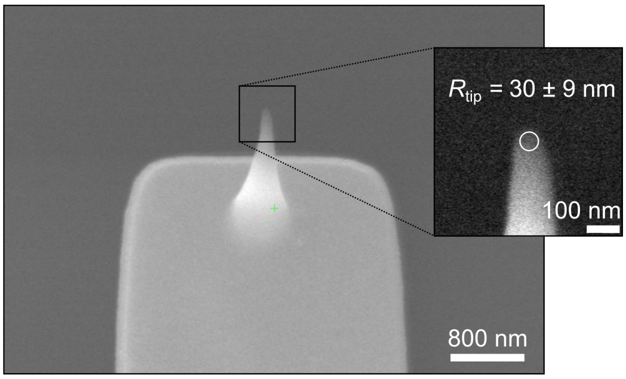

Supplementary Fig. 8 | Evaluation of the tip radius after AFM shear-force experiments | Representative SEM image of a cantilever and tip, along with a zoom to the tip itself (inset). New cantilevers were used for each experiment and subsequently imaged without coating in a low-pressure air environment. The mean tip radius was found to be 30 ± 9 nm (size of circle in inset).

The manufacturer's nominal radius of curvature for the cantilevers used is < 10 nm [26], but the tip's high density carbon/diamond-like carbon material may wear when subject to lateral forces in contact with a substrate. Experimentally, we found a mean tip radius of R_{tip} = 30 ± 9 (n=7) regardless of duration and numbers of force curves acquired in a set of experiment. We hence used this value in Eq. (1).

To quantify how this tip-wear affects the measurement itself –the magnitude of the recorded shear force, F_S– we tracked the evolution of F_S upon blunting of the tip. We used bilayers composed of DOPC and 30 mol% cholesterol to amplify any impact of the blunting on F_S, compared to pure DOPC. Since cholesterol reduces the diffusion of fluid-phase lipids in model membranes[3,6,10] but maintains a single phase liquid-disordered state[27]. We first recorded the deflection ($\propto F_N$) and the cantilever torsional response ($\propto F_S$) at the point the tip punctured the supported bilayer with a pristine tip at various frequencies (Suppl. Fig. 9a open symbols), which demonstrated the expected frequency dependencies. With the tip still immersed in the fluid, and with the sample still present, we then destructively blunted the tip by rapidly scanning the mica substrate (19.53 Hz line rate; 7 × 7 µm^2 scan area) with a large deflection setpoint (8.0 V) and low integral gain (4.0) for 20 frames to increase the effective tip radius

of curvature as much as possible. The shear force measurements were then repeated (in a different sample location, to avoid any debris from the blunting), with the same parameters (Suppl. Fig. 9a). The deflection at membrane puncture, and thus the force required to break through (black symbols) is approximately double that of the fresh tip, confirming that our protocol has indeed blunted the tip. Assuming that the pressure required to disrupt the membrane is constant, we can estimate that the tip-lipid contact area has also doubled due to the blunting. Despite this dramatic increase, the shear force (red symbols, upper panel) demonstrates very little change overall, implying that the measurement is (at this scale) robust to small changes in R_{tip}. This is to be expected from the Evans-Sackmann approximation[12,13] whereby we expect $F_{\mathrm{S}} \propto A_{\mathrm{tip}} \propto R_{\mathrm{tip}}\Delta h$. Assuming the membrane to behaves as a thin film supported by a hard substrate and indented by a spherical tip apex of radius R_{tip}, we expect the applied load force F_N normal to the membrane to satisfy $F_{\mathrm{N}} \propto R_{\mathrm{tip}}\ \Delta h^{\alpha}$, with $\alpha \geq 3/2$.[28] We can hence write: $F_{\mathrm{S}} \propto R_{\mathrm{tip}}\Delta h \propto {R_{\mathrm{tip}}}^{(1-\beta)}\ {F_{\mathrm{N}}}^{\beta}$ with $\beta = 1/\alpha$ so that $0 < \beta \leq 2/3$. Experimentally, we typically find $\beta \sim 0.5$. This confirms the sub-linear dependence of F_{S} on F_{N}, and hence the reduced impact of tip radius/contact area on the measurement.

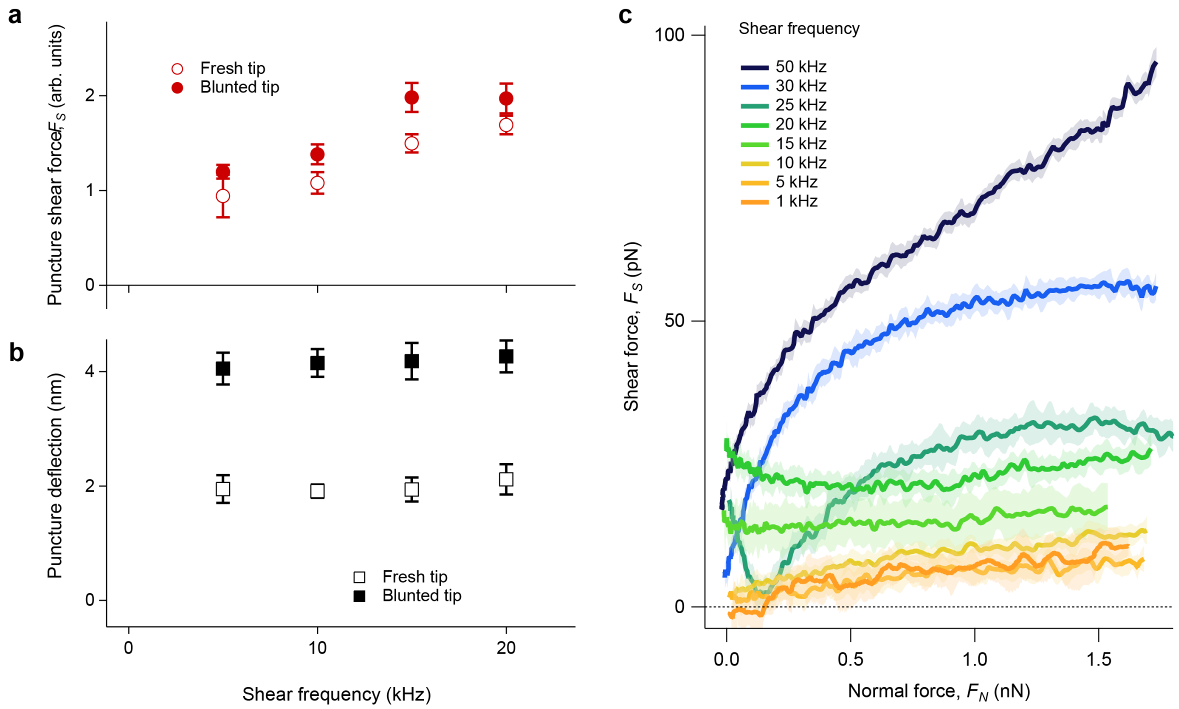

Supplementary Fig. 9 | Impact of the tip radius on the measured shear force at the point of membrane rupture. Comparison of the shear force (a) and normal load (through the lever deflection, (b)) at the point of membrane rupture for a fresh tip before (open symbols) and deliberately blunted tip (filled symbols). The blunting yields an approximate doubling of the force required to break through the bilayer thus implying a doubling of the contact area. Despite this, the shear force, F_{S}, does not change significantly after the blunting due to the sub-linear dependence of F_S on the normal load (c). The data in (c) is acquired in a DOPC bilayer from the same experiment used to produce Fig. 1. Each datapoint in the figure is the average of 15 individual measurements and the uncertainty represents the associated standard deviation. Source data are provided as a Source Data file.

Supplementary Note 5. Local membrane perturbation

The measurements on the bovine eye lens membrane in Fig. 4 of the main text clearly demonstrate the effect of its heterogeneous molecular organisation on the dynamic properties of each region. Being force-based, the measurement is necessarily perturbative, but by limiting the maximum normal force, we take care that the tip-sample interaction is elastic – that is, that there is no permanent deformation – while ensuring that sufficient signal in the lateral force channel is recorded.

In some circumstances, it may be of interest to choose to perturb the membrane, for example to probe the stability of the locally ordered proteins. To achieve this, the maximum force can be increased such that the stable regions are disrupted, as exemplified in Suppl. Fig. 10a-b, where a normal load of ≈1.2 nN is sufficient to puncture the membrane in the initial curves (c, lower), resulting in a transition towards lower lateral forces (c, upper) that relates to an increase in the apparent diffusion coefficient (d). However, quantification of the diffusion coefficient through Eq. (1) requires a priori knowledge of the diffusing species, here assumed lipids. This is not necessarily obvious in protein-rich regions.

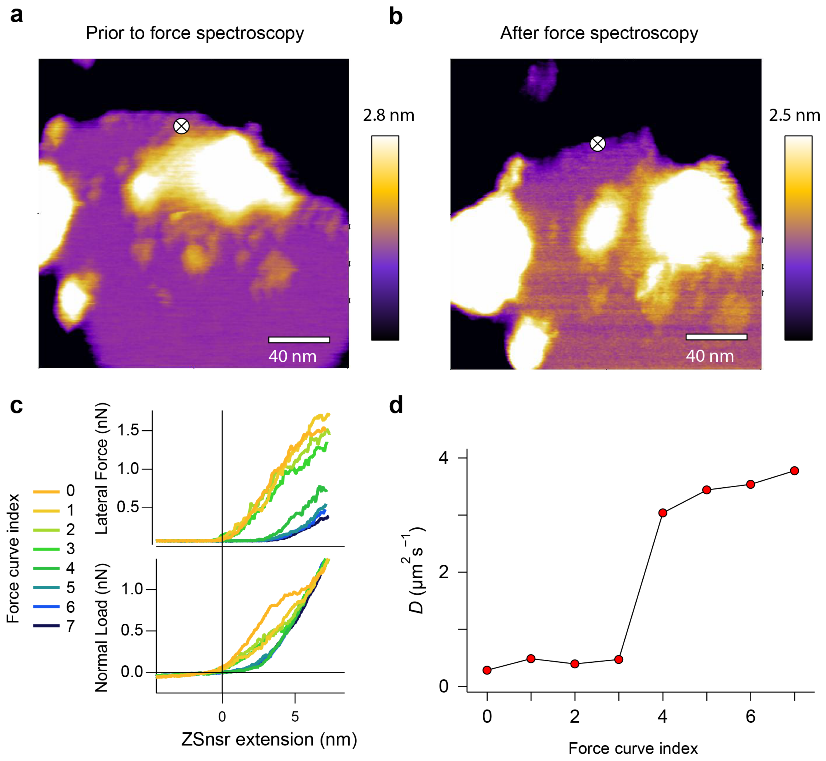

Supplementary Fig. 10: Dynamic rearrangements in native membranes | AM-AFM topographic scans prior to (a) and after (b) a series of force spectroscopy curves highlight the rearrangement of the native lens membrane in response to the perturbation. The spectroscopy curves themselves (c, taken in between scans a and b at the location marked by the ⊗ symbol) demonstrate the point at which this transition occurs, especially in the lateral force channel (between curve number 3 and 4). Following the same analysis as in the paper and assuming lipids as the main diffusing specie, a diffusion coefficient (d) is calculated for an indentation of 2.5 nm, highlighting changes in the local molecular arrangement of the membrane with a subsequent return to a highly diffusive regime. Applying Eq. (1) requires knowledge of the dominating diffusion specie. Source data are provided as a Source Data file.

Supplementary Methods' Figures and Table

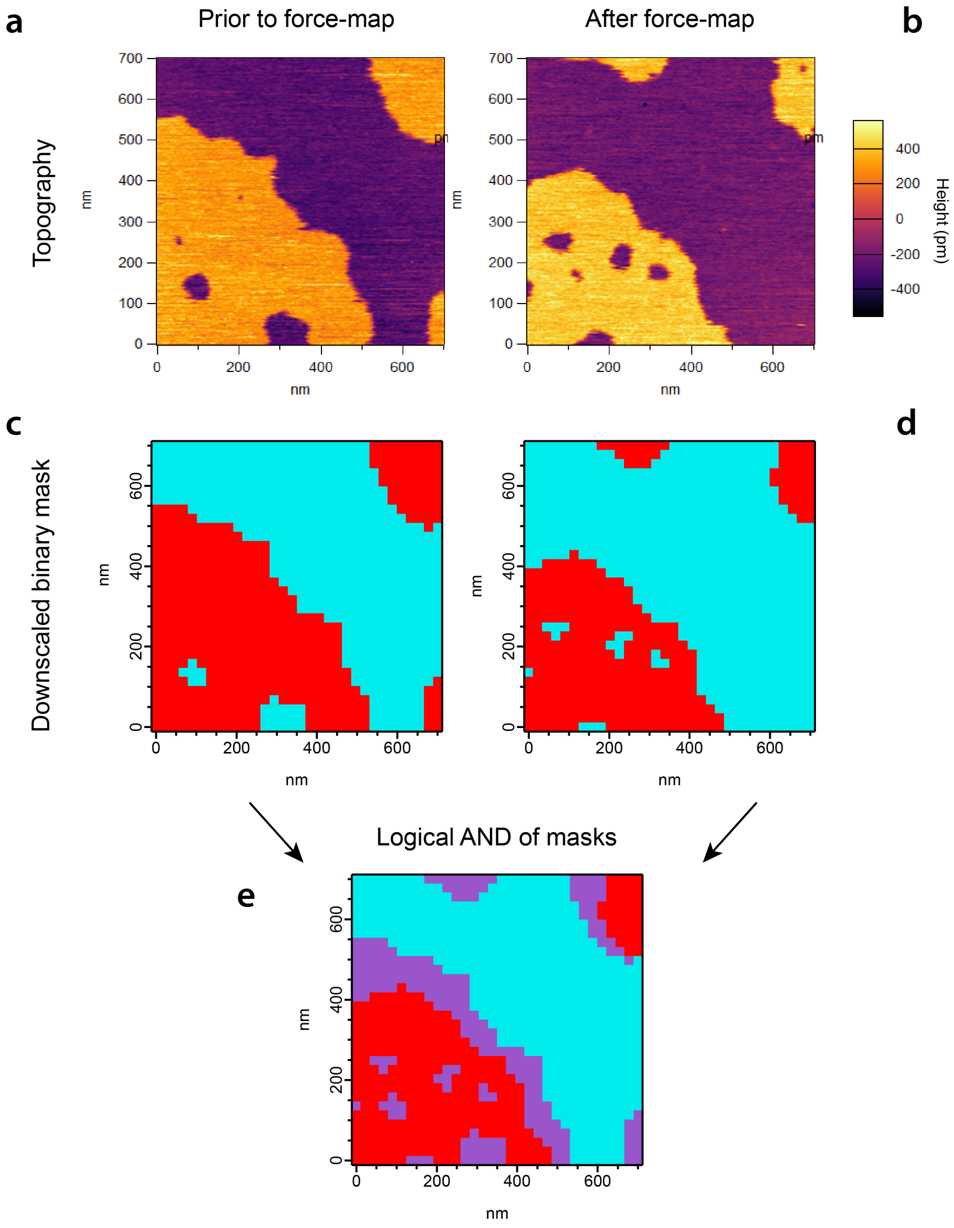

Supplementary Fig. 11 | Ascribing of spectroscopy curves to domains during mapping. When measuring membranes containing different types of domains, it is crucial to correctly ascribe any given force spectroscopy measurement to a particular domain. Over relatively long spectroscopy mapping measurements, instrumental drift may lead to ambiguous interpretation over border regions between different domains. To address this issue, topography images are acquired immediately prior (a) and after (b) the force map (Topography) followed by height thresholding (c-d) makes it possible to compare each domain before and after the map (Downscale binary, red and cyan). The boarder regions do not necessarily overlap (e, purple in Logical AND mask) due to drift and the measurement potential disturbing the membrane. Only curves corresponding to overlapping locations are retained.

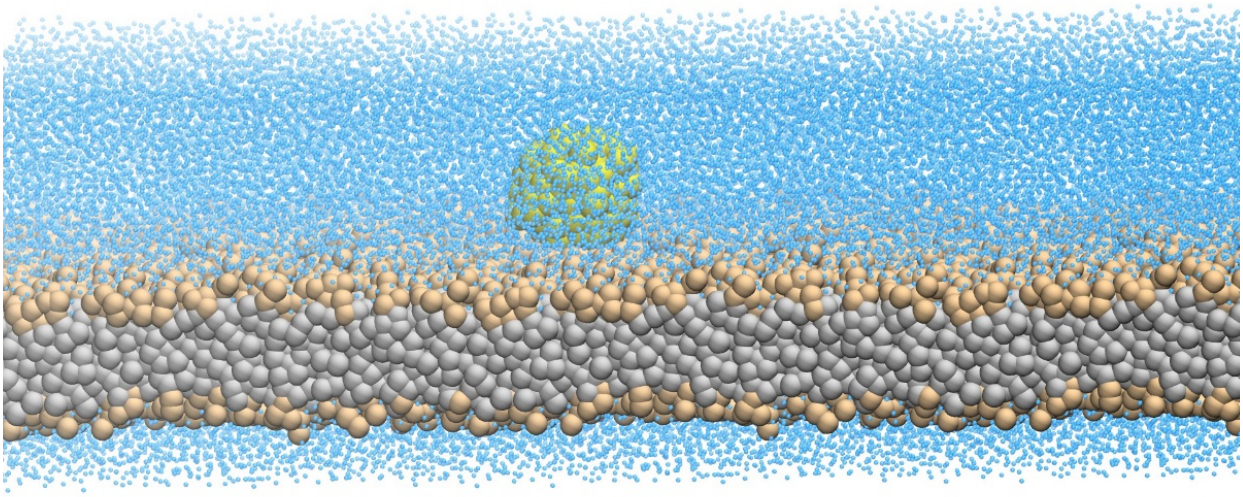

Supplementary Fig. 12 | The coarse-grained simulation box. Waters are shown in transparent blue and the remaining bead colouring is the same as in the main text. The silica sphere radius is 2 nm.

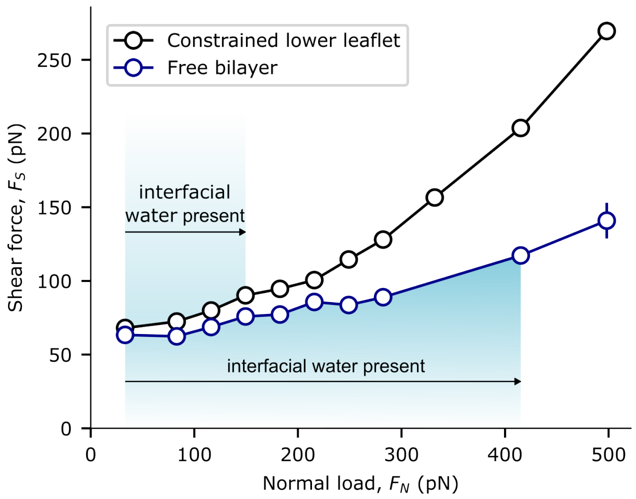

Supplementary Fig. 13 | Constrained membranes show different hydration and lubrication behaviours. Both free-standing bilayers and those with their lower leaflets constrained show similar behaviour at low normal loads. However, at high F_N (> 150 pN), the two systems diverge, as the interfacial water between the tip and the lipid headgroups of the constrained lipid bilayer is squeezed out and F_S dramatically increases (black). In contrast, for the free membrane (dark blue), the interfacial waters endure until $F_N \approx 400$ pN, presumably due to the bilayer's increased ability to deform in response to the load. As a result, the measured shear forces are substantially reduced.

Supplementary Table 2: Parameters for the conducted simulations. The folder ID refers to the folder where the corresponding simulations are freely available in the data supporting this publication

**Folder ID**	Simulation ID	Normal load (kJ mol^{-1} nm^{-1})	Tip radius (nm)	Shearing velocity (μm s^{-1})	Number of simulations	Simulation Length (ns)	Total simulation time (ns)	Fixed Lower Leaflets
**1**	1	20	2	5000	100	800	104000	Yes
	2			10000	50	400		
	3			50000	50	80		
	4			100000	50	40		
	5			200000	50	20		
	6			800000	50	8		
	7			1500000	50	4		
**2**	8	50	2	5000	100	800	81000	
	9			200000	50	20		
**3**	10	70	2	5000	100	800	81000	
	11			200000	50	20		
**4**	12	90	2	5000	100	800	81000	
	13			200000	50	20		
**5**	14	110	2	5000	100	800	104000	
	15			10000	50	400		
	16			50000	50	80		
	17			100000	50	40		
	18			200000	50	20		
	19			800000	50	8		
	20			1500000	50	4		
**6**	21	130	2	5000	100	800	81000	
	22			200000	50	20		
**7**	23	150	2	5000	100	800	81000	
	24			200000	50	20		
**8**	25	170	2	5000	100	800	81000	
	26			200000	50	20		
**9**	27	200	2	5000	100	800	81000	
	28			200000	50	20		
**10**	29	300	2	200000	50	20	1000	
**11**	30	400	2	200000	50	20	1000	
**12**	31	500	2	200000	50	20	1000	
**13**	32	600	2	200000	50	20	1000	
**14**	33	700	2	200000	50	20	1000	
**15**	34	800	2	200000	50	20	1000	
**16**	35	900	2	200000	50	20	1000	
**17**	36	1000	2	200000	50	20	1000	
**18**	37	20	2	5000	15	800	81000	No
	38			200000	50	20		
**19**	39	50	2	200000	50	20	1000	
**20**	40	70	2	200000	50	20	1000	
**21**	41	90	2	5000	15	800	81000	
	42			200000	50	20		
**22**	43	110	2	200000	50	20	1000	
**23**	44	130	2	200000	50	20	1000	
**24**	45	150	2	200000	50	20	1000	
**25**	46	170	2	200000	50	20	1000	
**26**	47	250	2	200000	50	20	1000	

Supplementary References

1. Tamm, L. K. & McConnell, H. M. Supported phospholipid bilayers. *Biophys. J.* **47**, 105–113 (1985).
2. Przybylo, M. *et al.* Lipid Diffusion in Giant Unilamellar Vesicles Is More than 2 Times Faster than in Supported Phospholipid Bilayers under Identical Conditions. *Langmuir* **22**, 9096–9099 (2006).
3. Benda, A. *et al.* How To Determine Diffusion Coefficients in Planar Phospholipid Systems by Confocal Fluorescence Correlation Spectroscopy. *Langmuir* **19**, 4120–4126 (2003).
4. Ratto, T. V. & Longo, M. L. Obstructed Diffusion in Phase-Separated Supported Lipid Bilayers: A Combined Atomic Force Microscopy and Fluorescence Recovery after Photobleaching Approach. *Biophys. J.* **83**, 3380–3392 (2002).
5. Zhang, L. & Granick, S. Lipid diffusion compared in outer and inner leaflets of planar supported bilayers. *J. Chem. Phys.* **123**, (2005).
6. Zhang, Y., Li, Q., Dong, M. & Han, X. Effect of cholesterol on the fluidity of supported lipid bilayers. *Coll. Surf. B* **196**, 111353 (2020).
7. Yan, R., Chen, K. & Xu, K. Probing Nanoscale Diffusional Heterogeneities in Cellular Membranes through Multidimensional Single-Molecule and Super-Resolution Microscopy. *J. Am. Chem. Soc.* **142**, 18866–18873 (2020).
8. Shim, S.-H. *et al.* Super-resolution fluorescence imaging of organelles in live cells with photoswitchable membrane probes. *Proc. Natl. Acad. Sci. U. S. A.* **109**, 13978–13983 (2012).
9. Woodward, X. & Kelly, C. V. Single-lipid dynamics in phase-separated supported lipid bilayers. *Chem. Phys. Lipids* **233**, 104991 (2020).
10. Filippov, A., Orädd, G. & Lindblom, G. The Effect of Cholesterol on the Lateral Diffusion of Phospholipids in Oriented Bilayers. *Biophys. J.* **84**, 3079–3086 (2003).
11. Chaikin, P. M. & Lubensky, T. C. *Principles of Condensed Matter Physics*. (Cambridge University Press, Cambridge, 2013).
12. Evans, E. & Sackmann, E. Translational and rotational drag coefficients for a disk moving in a liquid membrane associated with a rigid substrate. *J. Fluid Mech.* **194**, 553–561 (1988).
13. Kühner, M., Tampé, R. & Sackmann, E. Lipid mono- and bilayer supported on polymer films: composite polymer-lipid films on solid substrates. *Biophys. J.* **67**, 217–226 (1994).
14. Saffman, P. G. & Delbruck, M. Brownian motion in biological membranes. *Proc. Natl. Acad. Sci. U. S. A.* **72**, 3111–3113 (1975).

15. Marsh, D. *Handbook of Lipid Bilayers*. (CRC Press, 2013).

16. Moran, P. A. P. The Interpretation of Statistical Maps. *J. R. Stat. Soc. Ser. B Methodol.* **10**, 243–251 (1948).

17. Rey, S. J., Arribas-Bel, D. & Wolf, L. J. *Geographic Data Science with Python*. (CRC Press, Taylor & Francis Group, Boca Raton, 2023).

18. Ando, T., Uchihashi, T. & Kodera, N. High-Speed AFM and Applications to Biomolecular Systems. *Annu. Rev. Biophys.* **42**, 393–414 (2013).

19. Heath, G. R. & Scheuring, S. Advances in high-speed atomic force microscopy (HS-AFM) reveal dynamics of transmembrane channels and transporters. *Curr. Opin. Struct. Biol.* **57**, 93–102 (2019).

20. Ando, T., Uchihashi, T. & Fukuma, T. High-speed atomic force microscopy for nano-visualization of dynamic biomolecular processes. *Prog. Surf. Sci.* **83**, 337–437 (2008).

21. Shimizu, M. *et al.* An ultrafast piezoelectric Z-scanner with a resonance frequency above 1.1 MHz for high-speed atomic force microscopy. *Rev. Sci. Instrum.* **93**, 013701 (2022).

22. Shea, S. J. O. & Welland, M. E. Atomic Force Microscopy at Solid - Liquid Interfaces. *Langmuir* **7463**, 4186–4197 (1998).

23. Li, T.-D. & Riedo, E. Nonlinear Viscoelastic Dynamics of Nanoconfined Wetting Liquids. *Phys. Rev. Lett.* **100**, 106102 (2008).

24. Li, T.-D., Chiu, H.-C., Ortiz-Young, D. & Riedo, E. Nanorheology by atomic force microscopy. *Rev. Sci. Instrum.* **85**, 123707 (2014).

25. Cafolla, C., Foster, W. & Voïtchovsky, K. Lubricated friction around nanodefects. *Sci. Adv.* **6**, 1–9 (2020).

26. USC-F1.5-k0.6 - NanoWorld®. https://www.nanoworld.com/Ultra-Short-Cantilevers-USC-F1.5-k0.6.

27. Veatch, S. L. & Keller, S. L. Miscibility Phase Diagrams of Giant Vesicles Containing Sphingomyelin. *Phys. Rev. Lett.* **94**, 148101 (2005).

28. Garcia, P. D. & Garcia, R. Determination of the Elastic Moduli of a Single Cell Cultured on a Rigid Support by Force Microscopy. *Biophys. J.* **114**, 2923–2932 (2018).
